# Randomized crossover clinical trial of coenzyme Q10 and nicotinamide riboside in chronic kidney disease

**DOI:** 10.1172/jci.insight.167274

**Published:** 2023-06-08

**Authors:** Armin Ahmadi, Gwenaelle Begue, Ana P. Valencia, Jennifer E. Norman, Benjamin Lidgard, Brian J. Bennett, Matthew P. Van Doren, David J. Marcinek, Sili Fan, David K. Prince, Jorge Gamboa, Jonathan Himmelfarb, Ian H. de Boer, Bryan R. Kestenbaum, Baback Roshanravan

**Affiliations:** 1 Department of Medicine, Division of Nephrology, UCD, Davis, California, USA.; 2 Kinesiology Department, California State University, Sacramento, California, USA.; 3 Department of Radiology, University of Washington, Seattle, Washington, USA.; 4 Department of Internal Medicine, Division of Cardiovascular Medicine, UCD, Davis, California, USA.; 5 Department of Medicine, University of Washington, Seattle, Washington, USA.; 6 Obesity and Metabolism Research Unit, Western Human Nutrition Research Center, USDA, ARS, Davis, California, USA.; 7 Fred Hutchinson Cancer Research Center, Seattle, Washington, USA.; 8 Department of Biostatistics, UCD, Davis, California, USA.; 9 Department of Medicine, Division of Nephrology, Kidney Research Institute, University of Washington, Seattle, Washington, USA.; 10 School of Medicine, Vanderbilt University, Nashville, Tennessee, USA.

**Keywords:** Clinical Trials, Nephrology, Chronic kidney disease, Mitochondria, Skeletal muscle

## Abstract

**Background:**

Current studies suggest mitochondrial dysfunction is a major contributor to impaired physical performance and exercise intolerance in chronic kidney disease (CKD). We conducted a clinical trial of coenzyme Q10 (CoQ10) and nicotinamide riboside (NR) to determine their impact on exercise tolerance and metabolic profile in patients with CKD.

**Methods:**

We conducted a randomized, placebo-controlled, double-blind, crossover trial comparing CoQ10, NR, and placebo in 25 patients with an estimated glomerular filtration rate (eGFR) of less than 60mL/min/1.73 m^2^. Participants received NR (1,000 mg/day), CoQ10 (1,200 mg/day), or placebo for 6 weeks each. The primary outcomes were aerobic capacity measured by peak rate of oxygen consumption (VO_2_ peak) and work efficiency measured using graded cycle ergometry testing. We performed semitargeted plasma metabolomics and lipidomics.

**Results:**

Participant mean age was 61.0 ± 11.6 years and mean eGFR was 36.9 ± 9.2 mL/min/1.73 m^2^. Compared with placebo, we found no differences in VO_2_ peak (*P* = 0.30, 0.17), total work (*P* = 0.47, 0.77), and total work efficiency (*P* = 0.46, 0.55) after NR or CoQ10 supplementation. NR decreased submaximal VO_2_ at 30 W (*P* = 0.03) and VO_2_ at 60 W (*P* = 0.07) compared with placebo. No changes in eGFR were observed after NR or CoQ10 treatment (*P* = 0.14, 0.88). CoQ10 increased free fatty acids and decreased complex medium- and long-chain triglycerides. NR supplementation significantly altered TCA cycle intermediates and glutamate that were involved in reactions that exclusively use NAD^+^ and NADP^+^ as cofactors. NR decreased a broad range of lipid groups including triglycerides and ceramides.

**Conclusions:**

Six weeks of treatment with NR or CoQ10 improved markers of systemic mitochondrial metabolism and lipid profiles but did not improve VO_2_ peak or total work efficiency.

**Trial registration:**

ClinicalTrials.gov NCT03579693.

**Funding:**

National Institutes of Diabetes and Digestive and Kidney Diseases (grants R01 DK101509, R03 DK114502, R01 DK125794, and R01 DK101509).

## Graphical abstract

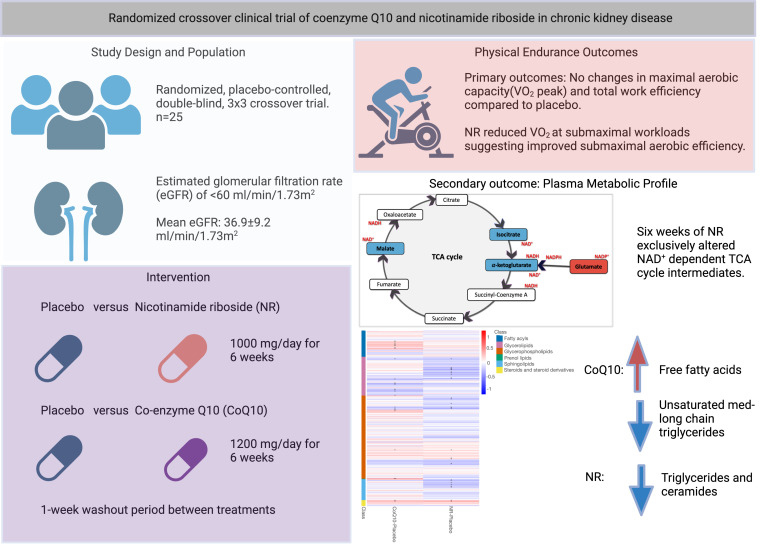

## Introduction

Patients with chronic kidney disease (CKD) suffer from a loss of functional independence and frailty (1, 2). Comorbid illnesses associated with CKD undoubtedly play a role in impaired muscle function and physical endurance (3). In addition, several lines of evidence suggest that kidney dysfunction itself contributes directly to a reduction in skeletal muscle function and mass (4). Among the mechanisms under investigation (5), disruption of mitochondrial oxidative capacity is considered to be a central candidate linking kidney disease with skeletal muscle impairment (2, 6). Interventions that target mitochondrial function in CKD have not been evaluated in human studies.

Coenzyme Q10 (CoQ10) is a fat-soluble coenzyme involved in the transfer of electrons from complex I and II to complex III in the mitochondria during oxidative phosphorylation (7). Deficiency in CoQ10 levels has been associated with oxidative stress and impaired mitochondrial function in CKD (8). Plasma concentration of CoQ10 is reduced in patients with CKD compared with healthy controls (9); however, little is known about its treatment effects on mitochondrial metabolism, systemic inflammation, and physical performance. CoQ10 supplementation studies in aged mice have shown enhanced sirtuin 1 (SIRT1) and peroxisome proliferator-activated receptor-γ coactivator-1α (PGC-1α )expression, leading to improved mitochondrial function and inhibition of oxidative stress (10). Improvements in β oxidation of fatty acids and suppressed lipid accumulation have also been reported in diabetic obese mice (11). Among patients with kidney failure, CoQ10 administration has been shown to reduce markers of oxidative stress (9).

Nicotinamide riboside (NR), a precursor to NAD^+^, is a crucial cofactor and an electron carrier involved in oxidative metabolism, mitochondrial biogenesis, and redox homeostasis (12). CKD is associated with reduced NAD^+^ biosynthesis and increased consumption limiting NAD^+^ bioavailability (13). NAD^+^ precursor supplementation may have the potential to target metabolic and clinical complications associated with CKD, including dyslipidemia, skeletal muscle dysfunction, impaired amino acid metabolism, and oxidative stress (14, 15). Indeed, administration of NR has been shown to improve insulin sensitivity in obese mice, increase NAD^+^ levels leading to augmented mitochondrial SIRT3 activity, and improve muscle endurance along with muscle mitochondrial content (16, 17). Despite the promising results from both rodent and human studies, the impact of NAD^+^ supplements on systemic mitochondrial function and physical performance CKD are still lacking.

Given the central role of mitochondrial dysfunction in the pathogenesis of kidney related skeletal muscle dysfunction (18), we hypothesized that therapies targeting muscle mitochondrial metabolism could improve physical endurance and systemic markers of mitochondrial metabolism in CKD. We conducted a randomized, placebo-controlled, crossover trial to test the impact of CoQ10 and NR on maximal and submaximal physical endurance capacity, work efficiency, and metabolic profiles in patients with CKD.

## Results

### Participant characteristics.

A total of 25 participants were enrolled and completed the study (Supplemental Figure 1; supplemental material available online with this article; https://doi.org/10.1172/jci.insight.167274DS1). The mean age of the cohort was 61.0 ± 11.6 years and 40% were female. The mean eGFR was 36.9 ± 9.2 mL/min/1.73 m^2^, and 16% of the participants had a diagnosis of diabetes. The median self-reported physical activity of the participants at enrollment was 13 hours per month (IQR [2–30]), and all participants had an Activities of Daily Living (ADL) score of 8 except for 1 participant with an ADL of 7. The average hemoglobin among participants was 12.7 ± 1.8 gm/dL. Characteristics of the participants in this study can be found in Table 1.

### Six weeks of NR or CoQ10 treatment did not impact physical endurance and cardiorespiratory fitness outcomes compared with placebo.

**Figure 1:**
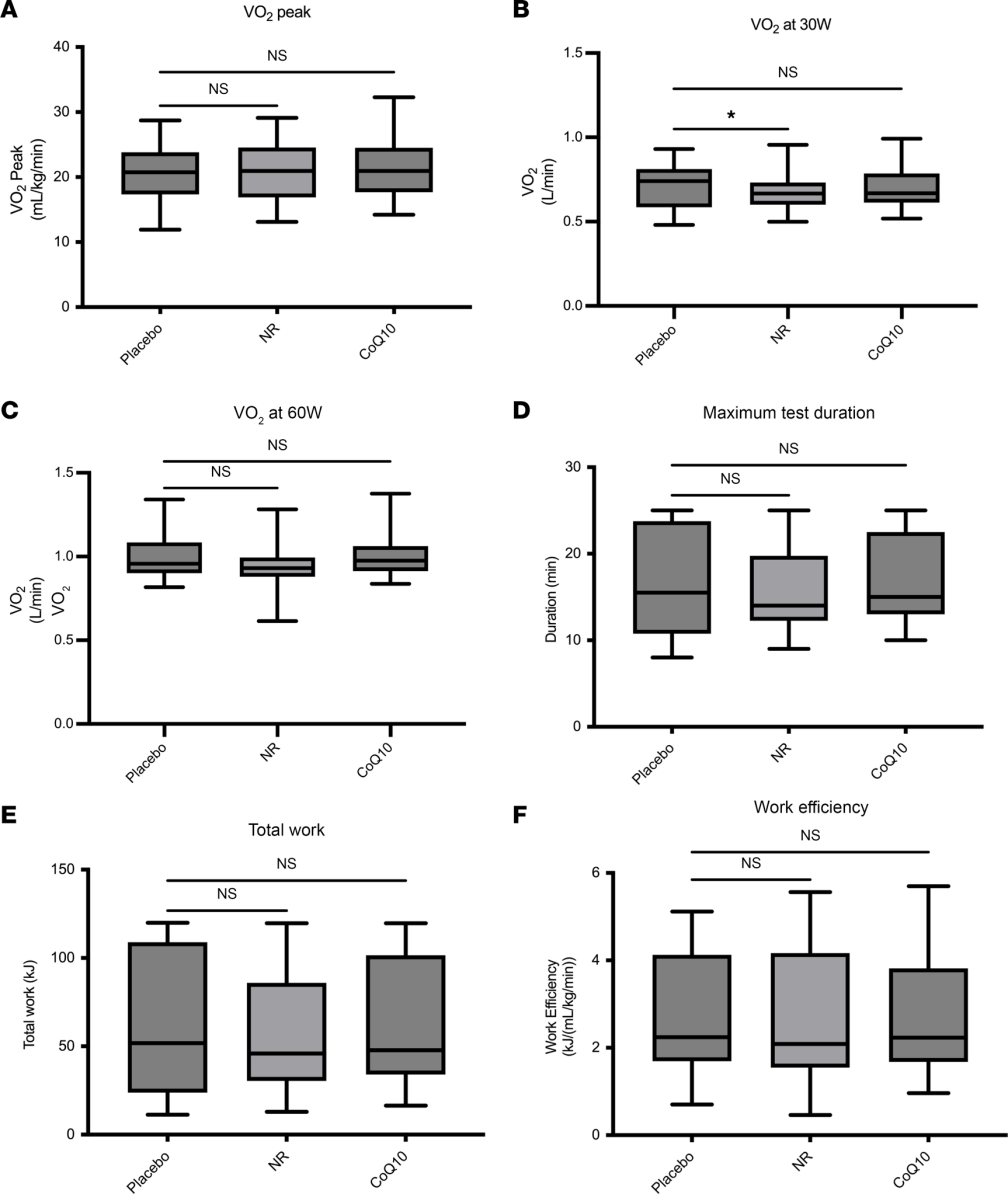
No changes in physical endurance and CRF outcomes after NR and CoQ10 treatments compared with placebo (*n* = 25). (**A**) VO_2_ peak, (**B** and **C**) absolute VO_2_ at 30 and 60 W, (**D**) test duration, (**E**) total work, and (**F**) total work efficiency. Two-way ANOVA was used to compare changes in response to NR and CoQ10 with placebo. The box plots represent median and IQR and the whiskers represent minimum and maximum values. *P < 0.05. NS, not significant.

The mean VO_2_ peak after placebo was 20.64 ± 4.84 mL/kg/min. Cardiorespiratory fitness (CRF) and VO_2_ peak were not significantly different with NR and CoQ10 supplementation, with means of 21.38 ± 4.93 mL/min/kg (*P* = 0.36) and 21.41 ± 4.74 (*P* = 0.33), respectively (Figure 1A and Table 2). Submaximal efficiency assessed by VO_2_ at a given submaximal workload was affected by NR treatment. Mean submaximal absolute VO_2_ after placebo was 0.70 ± 0.12 L/min at 30 W and 0.99 ± 0.12 L/min at 60 W. Compared with placebo, NR reduced submaximal absolute VO_2_ at both 30 W to a mean of 0.67 ± 0.1 L/min (*P* = 0.03) and 60 W to a mean of 0.95 ± 0.14 L/min (*P* = 0.07) (Figure 1, B and C, and Table 2). However, CoQ10 did not impact submaximal efficiency at 30 W and 60 W (Figure 1, B and C, and Table 2). Mean duration of cycle ergometry after NR and CoQ10 supplementation was 982 ± 317 and 1,012 ± 332, respectively, compared with 1,009 ± 332 seconds with placebo (Figure 1D and Table 2). Physical endurance measured as total work performed during cycle ergometry did not change after NR, with a mean of 57.72 ± 37.29 kJ (*P* = 0.47) and after CoQ10 with a mean of 61.5 ± 38.35 (*P* = 0.77) compared with placebo with a mean of 60.32 ± 39.06 (Figure 1E and Table 2). NR and CoQ10 supplementation did not have an impact on total work efficiency, with means of 30.9 ± 18.1 kJ/(L/min) (*P* = 0.46) and 33.3 ± 15.7 (*P* = 0.55) compared with placebo with a mean of 32.2 ± 17.5 (Figure 1F and Table 2). There were no alterations in fuel utilization at rest indicated by no change in respiratory exchange ratio (RER) at rest (*P =* 0.41 and 0.62) and VO_2_ max (*P* = 0.44 and 0.87) after NR and CoQ10. However, RER at 30 W and 60 W were increased after NR (*P =* 0.06 and 0.04) compared with placebo (Table 2). CoQ10 did not have an impact on RER at 30 W or 60 W compared with placebo (*P* = 0.24 and 0.18) (Table 2). Estimates of differences in physical endurance outcomes, BP, and BW changes comparing NR and CoQ10 to placebo are shown in Supplemental Table 1. Analyses of treatment by study period revealed no evidence for carryover effects among the study interventions.

In the subgroup analysis, CRF (relative VO_2_ peak, mL/kg/min) was higher and RER at 60 W was significantly lower among participants classified as active versus sedentary and good versus poor performers (Supplemental Figure 2, A–D). However, we found no evidence for an impact of NR or CoQ10 on CRF or physical endurance (total work and work efficiency) in these subgroups (Supplemental Figure 2, A and B). Similarly, we found no meaningful or significant impact of NR or CoQ10 supplementation on RER at 60 W in our subgroup analysis (Supplemental Figure 2, C and D). Gender-stratified analysis showed no differential improvements in VO_2_ peak and RER at 60 W among males and females (Supplemental Figure 2, E and F).

### NR or CoQ10 treatment did not change kidney function and inflammatory biomarkers compared with placebo.

Over the 6-week treatment period, we found no meaningful or significant change in BW (Supplemental Table 1) or serum triglyceride associated with NR and CoQ10 supplementation compared with placebo (*P =* 0.51 and 0.69, and *P =* 0.71 and 0.70, respectively). We found no meaningful or statistically significant differences in the urine albumin-to-creatinine ratio (UACR) (P = 0.28 and 0.21) (Supplemental Figure 3A), kidney function biomarkers, including serum creatinine (*P =* 0.23 and 0.58) and cystatin C (*P =* 0.08 and 0.87) (Supplemental Figure 3, B and C), and inflammatory biomarker serum C-reactive protein (CRP) (*P* = 0.21 and 0.12) (Supplemental Figure 3D) after NR and CoQ10, respectively.

### CoQ10 supplementation increased plasma free fatty acid and decreased triglycerides.

Three of the 98 tested plasma metabolites decreased in response to CoQ10 treatment compared with placebo at the 0.05 significance level: β-alanine, lactate, and 2-deoxytetronic acid with fold changes of 0.49 (*P* = 0.01), 0.72 (*P* = 0.02), and 0.79 (*P* = 0.03), respectively (data not shown). In contrast, CoQ10 had an impact on the plasma lipid profile, altering 6% (24/394) of the detected lipid species compared with placebo (Supplemental Table 2). These changes predominantly involved free fatty acids (FFAs) (8/24) and triglycerides (7/24). All of the altered FFAs were increased compared with placebo, ranging from an increase of 19% to 40%. Triglycerides showed the opposite pattern with a systematic decrease compared with placebo, ranging from a decrease of 19% to 66% (Supplemental Table 2). The altered triglycerides tended to be long chained with a high degree of unsaturation. Plasma 3-hydroxybutarate was increased (not significantly), with a fold change of 1.41 (*P* = 0.07) compared with placebo (data not shown).

### NR treatment altered NAD^+^-dependent TCA cycle intermediates while decreasing plasma triglycerides and ceramides.

Compared with placebo, NR supplementation altered 6% (6/98) of the detected metabolites (Table 3). These metabolites included 3 a priori–targeted TCA cycle intermediates: isocitrate, α-ketoglutarate, and malate, which all are involved in reactions that use NAD^+^ as a cofactor. We found a significant decrease in all 3 TCA cycle intermediates compared with placebo (Table 3). In addition, glutamate, a precursor to α-ketoglutarate that feeds into the TCA cycle using NADP^+^ as a cofactor, was increased compared with placebo (Table 3). We confirmed a small to medium magnitude of difference on TCA cycle intermediates with effect sizes ranging from –0.37 (95% CI –0.06 to –0.62) (malate) to –0.40 (95% CI 0.1 to –0.71) (isocitrate) after NR supplementation (Table 3).

NR supplementation also had a meaningful impact on the lipid profile, significantly altering 7.5% (30/394) of detected lipid species (Supplemental Table 3). Lipid species were generally decreased with 26 out of 30 altered lipids reduced compared with placebo. We found a significant decrease in all 10 altered triglycerides. These triglycerides tended to be medium chained with a lower degree of unsaturation (Supplemental Table 3). The largest decrease was detected in triglyceride 50:0 with a 52% reduction compared with placebo. Reductions in ceramides, lyso-phosphatidylethanolamine (LPE), lyso-phosphatidylcholines (LPC), and phosphatidylethanolamines (PE) were also detected compared with placebo (Supplemental Table 3).

### Six weeks of CoQ10 or NR supplementation was associated with significant alterations in plasma fatty acyls (fatty acids), glycerolipids (triglycerides and diacylglycerides), glycerophospholipids (PEs, LPEs, LPCs, and PCs), and sphingolipids (ceramides and glucosylceramides).

**Figure 2:**
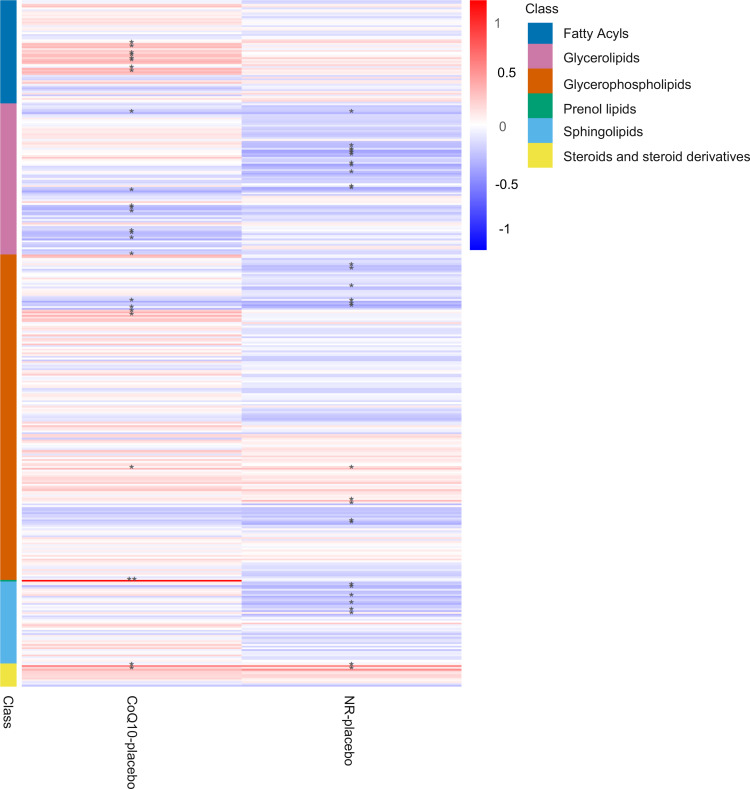
Heatmap depicting compositional changes within lipid classes in response to NR and CoQ10 supplementation. The colors of the heatmap are based on effect size obtained from linear mixed effects modeling adjusted for fasting status. *P < 0.05, **P < 0.001. The lipid classes include fatty acyls (fatty acids), glycerolipids (triglycerides and diacylglycerols), glycerophospholipids (PEs, LPEs, LPCs, and PCs), sphingolipids (ceramides and glycosylceramides), steroids, and steroid lipids (cholesterol and cholesteryl ester).

To investigate systemic plasma lipid profile changes in response to NR and CoQ10, we assessed compositional changes within major lipid classes compared with placebo. We found a systematic increase in fatty acyls and a decrease in glycerolipids after CoQ10 treatment compared with placebo (Figure 2). In comparison, NR supplementation was associated with a systemic decrease in glycerolipids and sphingolipids compared with placebo. NR also decreased plasma glycerophospholipids with the exception of 2 PC species (Figure 2 and Supplemental Table 3).

### Oral supplementation with 1,000 mg/day of NR and 1,200 mg/day of CoQ10 was well tolerated and elicited no serious adverse effects.

Twenty-three of the 25 participants consumed greater than 75% of NR, CoQ10, and placebo pills administered. We observed no difference in treatment-associated adverse events compared with placebo (Supplemental Table 4). During the trial, a total of 13 adverse events were reported by the participants, with 6 during NR, 3 during CoQ10, and 4 during placebo supplementation. The adverse events were counted from the start of the treatment until the end of the washout period.

## Discussion

In a randomized crossover trial testing 6 weeks of NR and CoQ10 treatments in patients with CKD, neither therapy improved the primary study outcomes of physical endurance measured by maximal aerobic capacity (VO_2_ peak) and total work efficiency. Exploratory analysis, however, revealed that 6 weeks of NR improved submaximal exercise efficiency by reducing absolute VO_2_ at 30 W and 60 W with a concomitant increase in RER, suggesting better efficiency in carbohydrate utilization at submaximal intensity. NR and CoQ10 treatments led to mechanistically plausible impacts on TCA cycle intermediates and lipid metabolites: CoQ10 increased plasma FFAs and decreased highly unsaturated medium- and long-chain triglycerides, and NR significantly altered plasma metabolites that are involved in reactions that use NAD^+^ or NADP^+^ as a cofactor, including 3 TCA cycle intermediates and glutamate. In addition to changes in plasma metabolites, NR supplementation resulted in a reduction in a wide range of lipid species, including triglycerides, ceramides, LPEs, LPCs, and PEs, compared with placebo. The treatments were generally well tolerated over the duration of the study. The observed impacts on the metabolic profile suggest early beneficial changes in systemic mitochondrial metabolism and lipid profile that argue for future trials with a longer treatment duration.

Our findings are consistent with previous studies that also show no physical performance-enhancing effect from short-term CoQ10 or NR supplementation across a wide range of ages and physical fitness levels. A prior study of NR supplementation involving older males showed improved skeletal muscle NAD^+^ levels and a reduction in circulating inflammatory cytokines after 21 days but no changes in grip strength or mitochondrial bioenergetics (19). Similarly, a 14-day CoQ10 supplementation among trained and untrained individuals showed reduction in plasma oxidative stress levels but no changes in anaerobic or aerobic capacity and ventilatory threshold (20). Negligible changes in metabolic substrate use indicated by comparable RER measurements at resting and 60 W after CoQ10 supplementation are also in agreement with previous findings. A randomized trial of patients with myalgia showed no changes in RER after 8 weeks of CoQ10 supplementation (21).

Contrary to previous studies, we detected changes in VO_2_ and RER at submaximal workloads (22, 23) in our post hoc exploratory analyses. In particular, we detected increased RER at 30 W and 60 W, suggesting enhanced carbohydrate utilization after NR supplementation. Higher submaximal RERs coincided with lower absolute VO_2_ at submaximal workload, suggesting improved submaximal exercise efficiency. It is known that the NAD^+^/NADH ratio regulates oxidative metabolism through sirtuins (24). A possible explanation for the enhanced carbohydrate utilization with NR supplementation is improved SIRT1 activity that directly regulates mitochondrial function and biogenesis through PGC-1α (25). Studies have shown that NAD^+^ supplementation modulates SIRT1 activity (16, 26, 27), leading to improved glucose utilization via improved oxidative metabolism. A long-term (5 months) NR supplementation study among BMI-discordant twins showed increased muscle mitochondrial biogenesis linked to upregulation of *SIRT1*, *TFAM*, *MFN2*, and *NRF1* — all genes involved in glucose metabolism (27). The changes in multiple TCA cycle intermediates may support improved efficiency of energy metabolism underlying the increased submaximal RER with NR supplementation. This suggests that short-term NR supplementation may lead to improved energy expenditure from carbohydrates without impacting fat oxidation during light- to moderate-intensity exercise in CKD; however, further studies confirming this observation are needed. Together, our data along with published studies show that solely targeting mitochondrial dysfunction with short-term pharmacologic interventions does not counteract CKD-associated exercise intolerance. The complementary effects of CoQ10 and NR on lipid metabolism motivates future studies testing the combination of these supplements added to a structured exercise program to improve exercise tolerance in patients with CKD.

CoQ10 supplementation in our study was associated with metabolic changes suggesting improved mitochondrial β oxidation known to be disrupted in CKD. CKD is associated with adverse metabolic complications, including dyslipidemia (28), amino acid and protein catabolism (29), and insulin resistance (30), contributing to impaired mitochondrial energy metabolism (14, 31). Prior studies have demonstrated CoQ10 supplementation reduces lipid peroxidation, a maker of oxidative stress, in patients with kidney failure (32). Impaired mitochondrial β oxidation and consequent lipid accumulation contributes to inflammation, oxidative stress, and insulin resistance in CKD (14, 33). Several lines of evidence suggest CoQ10 favorably impacts β oxidation. First, we observed biologically relevant alterations in the structure and amount of plasma FFAs and triglycerides after CoQ10 supplementation (Supplemental Table 2 and Figure 2). Resting-state plasma FFA concentration is directly linked to the peak fat oxidation rate (34, 35). Further studies are needed to assess whether these changes in resting FFA reflect changes in insulin sensitivity. Second, CoQ10 supplementation resulted in a systemic decrease of medium- and long-chain triglycerides that accumulate in CKD and are hallmarks of impaired β oxidation (36). This also confirms previous studies showing reduction of serum triglycerides in response to CoQ10 supplementation (37). The reductions in longer triglycerides with high numbers of double bonds with CoQ10 treatment in our study suggests improved β oxidation. Finally, while not statistically significant (*P* = 0.07), there was a meaningful 41% increase in the plasma level of an important ketone body elevated in fatty acid oxidation, 3-hydroxybutyrate levels, a marker for β oxidation rate (38), with CoQ10. Our findings are consistent with prior in vitro and in vivo studies in mice showing improved β oxidation after CoQ10 supplementation (11, 39, 40). Our results build on findings from previous studies confirming that CoQ10 supplementation improves the lipid profile in CKD by increasing β oxidation and decreasing plasma medium- and long-chain triglycerides. These findings motivate future trials involving longer durations of CoQ10 supplementation investigating long-term improvements in fatty acid oxidation and reduction in medium- and long-chain triglycerides. These future studies have clinical implications for dyslipidemia management in CKD and alleviating its metabolic and physiological complications such as insulin resistance, impaired energy metabolism, and oxidative stress.

We found evidence that NR supplementation changed plasma levels of our targeted TCA cycle intermediates, suggesting improved systemic mitochondrial metabolism. Sufficient NAD^+^ levels are needed for a wide range of anabolic and catabolic pathways, including glycolysis, TCA cycle, oxidative phosphorylation, fatty acid oxidation, and pentose phosphate pathway (41). Diminished NAD^+^ biosynthesis results in depleted NAD^+^ levels in CKD, contributing to impairment in energy-producing metabolic pathways (13, 42). We found significant alterations in 3 targeted TCA cycle intermediates (isocitrate, α-ketoglutarate, and malate) that exclusively use NAD^+^ as a cofactor. In addition, glutamate, which is an anapleurotic precursor of α-ketoglutarate that uses NADP^+^ as a cofactor, was also significantly altered. Our results in patients with CKD agree with prior studies of NR supplementation in murine models of aging. A study using muscle stem cells from aged mice showed that NR supplementation increases the expression of genes associated with TCA cycle and oxidative phosphorylation in addition to increased oxidative respiration, mitochondrial membrane potential, and ATP production (43). Further evidence for an impact of NR on mitochondrial function comes from a recent study of 12-week NR supplementation in heart failure patients with reduced ejection fraction, showing an increased mitochondrial respiration and decreased proinflammatory cytokine expression in PBMCs (44). Future studies are needed to assess mitochondrial metabolism impairments associated with reduced NAD^+^ and the impact of NAD^+^ precursor supplementation on ex vivo mitochondrial bioenergetics and oxidative phosphorylation capacity in CKD.

NR treatment led to changes in the lipidomic profile, decreasing a broad range of plasma lipid species including triglycerides. CKD is known to be associated with disordered lipid metabolism with lower kidney function and greater albuminuria associated with impairments in β oxidation and higher levels of ceramides, triglycerides, and phosphatidylcholines (33, 45, 46). These lipid class alterations have been associated with biologic pathways of oxidative stress, insulin resistance, and inflammation in patients with CKD (47–49). NAD^+^ is a crucial cofactor needed for β oxidation of fatty acids. NAD^+^ deficiency in CKD contributes to impaired fatty acid β oxidation resulting in intracellular accumulation of lipids, including enrichment of triglycerides high in polyunsaturated fatty acids, which are thought to upregulate de novo biosynthesis of triglycerides and phospholipids via increased mitochondrial glycerol-3-phosphate acyltransferase (mtGPAT) signaling (36, 50, 51). Although total triglyceride levels remained unchanged with NR supplementation, there was evidence of metabolically favorable changes, particularly in reductions in subclasses of triglycerides that were predominantly short and medium chained with a high degree of saturation. Consistent with another prior study of NAD^+^ precursors in humans (44), our findings suggest that NR supplementation improved lipid metabolism by enhancing catabolism of shorter more saturated triglycerides without a significant impact on longer polyunsaturated triglycerides. However, future studies are needed to better understand the underlying mechanism of NAD^+^ supplementation on improved triglyceride metabolism.

In addition to reductions in triglycerides, NR treatment led to reductions in biologically relevant ceramides (a sphingolipid), LPEs, LPCs, and PEs (glycerophospholipids). The presence and severity of kidney disease is associated with increased ceramide levels known to adversely impact metabolic health (52). A recent large prospective cohort study showed that PEs, triglycerides, and ceramides are strongly associated with increased risk of CKD (53). Ceramides are considered lipotoxic, promoting lipid accumulation, insulin resistance, mitochondrial dysfunction, impaired β oxidation, inflammation, and apoptosis (54–56). Similarly, elevated LPE, LPC, and PE levels have also been implicated in cardiovascular disease, diabetes, and neurodegenerative disease (57–59). In our study, NR supplementation improved the plasma lipid profile by reducing lipotoxic species in CKD. Future studies are needed to investigate the underlying mechanism of improved CKD-associated dyslipidemia with increased NAD^+^ bioavailability and its long-term effect on kidney function and other clinical outcomes. Key candidates for improved mitochondrial and lipid metabolism are NAD^+^-dependent mitochondrial sirtuins (and their downstream effectors) involved in mitochondrial biogenesis, mitochondrial dynamics, fatty acid oxidation, and oxidative stress defense (60).

This study had notable strengths and limitations. First, we used an efficient and rigorous double dummy, placebo-controlled, randomized crossover trial design and physiologically relevant measures of endurance exercise capacity and substrate utilization during graded cycle ergometry testing. Second, we applied semitargeted metabolomics and lipidomics profiling and accounted for fasting status changes to identify changes in mitochondrial and lipid metabolism after NR and CoQ10 supplementation. However, this study had several limitations. First, the sample size was small and treatment duration was short, limiting evaluation to early potential treatment effects. This may have limited our ability to detect differences in muscle and exercise tolerance requiring longer-term treatment. Second, our study partially coincided with the start of the COVID-19 pandemic, leading to inevitable lifestyle changes among participants during the study period. We were unable to reliably track the impact this may have had on habitual physical activity. Third, a number of study participants were, on average, more active than the general CKD population who are, on average, much more sedentary (61). Fourth, we did not account for multiple comparisons in our lipidomics analyses. Nevertheless, our exploratory lipidomics analysis detected meaningful effects of CoQ10 and NR supplementation on specific biologically relevant functional classes of lipids known to be associated with CKD and demonstrated to have adverse effects on the kidney function. Finally, we did not have intracellular or tissue-specific (i.e., skeletal muscle) readouts of NAD^+^ or CoQ10 before and after NR or CoQ10 supplementation to confirm higher intracellular levels of NAD^+^ or CoQ10. Nonetheless, we did confirm a significant increase in plasma CoQ10 levels through our lipidomics analysis with an effect size of 1.3 (95% CI of 0.92 to 1.72) (*P* = 1.44 × 10^–11^) compared with placebo. Plasma and skeletal muscle CoQ10 levels have been shown to have a significant correlation in previous studies (62). Similarly, an NR supplementation study among older adults (70–80 years old) showed that 1,000 mg daily supplementation of NR for 21 days significantly elevated NAD^+^ metabolome in the skeletal muscle (19).

In contrast to the benefits of exercise in patients with CKD, our findings demonstrate NR or CoQ10 supplementation alone does not lead to improved maximal physical performance. Clinical trials of exercise training in persons with nondialysis CKD have shown significant improvements in maximal aerobic capacity, physical performance, and physical functioning (63, 64). Future studies combining exercise with these treatments are needed to detect synergy in improvement of metabolic health and exercise tolerance in patients with CKD given the overlapping mechanisms of exercise and NAD^+^ precursor supplementation. Animal studies demonstrate NAD^+^-mediated mitochondrial hormetic response (mitohormesis), a well-known mediator of exercise-induced adaptations (65), through activation of SIRT1 and SIRT3 modulating mitochondrial fitness following NAD^+^ precursor supplementation (66, 67). In addition, a prior clinical trial in elderly adults combining 4 months of an antioxidant shown to induce SIRT1 and mitochondrial biogenesis (68) with exercise training demonstrated increases in muscle strength and physical endurance via 6-minute walking distance compared with exercise alone (69). Further human studies are needed to investigate the signaling systems involved in regulation of mitochondrial homeostasis via NAD^+^ precursor supplementation and if they enhance mitohormetic effects improving adaptation to exercise training in CKD.

In conclusion, short term CoQ10 and NR supplementation in patients with moderate to severe CKD resulted in biologically plausible changes in mitochondrial metabolism and the plasma lipid profile. Metabolic changes were more pronounced for the plasma lipidome than the metabolome. CoQ10 treatment led to improved β oxidation resulting in a systemic increase in plasma FFAs and a decrease in complex triglycerides while NR supplementation altered levels of TCA cycle intermediates and resulted in a broad decrease of plasma lipid species, including lipotoxic subclasses of sphingolipids. These findings add to the body of preclinical evidence supporting the efficacy of NR and CoQ10 for improving markers of mitochondrial metabolism and the lipid profile in persons with CKD not treated with dialysis. Given the distinct beneficial impacts of NR and CoQ10 on plasma metabolome and lipid profile, future studies of longer duration are needed to investigate the potential synergistic effects of a combination therapy of NR and CoQ10 in sedentary patients with CKD.

## Methods

### Study design and procedures

CoQ10 and NR in CKD (CoNR trial) is a placebo-controlled, double-blind, randomized crossover trial with 3 arms: placebo, CoQ10, and NR (Clinicaltrials.gov NCT03579693). The trial was conducted from November 2018 to April 2021. There were 3 phases in the study, each 42 (6 weeks) days separated by a 1-week washout period. Subjects received 1,000 mg/day of NR (Niagen) or 1,200 mg/day of CoQ10 (Tishcon) for 6 weeks (Supplemental Figure 1). The doses and study duration were chosen based on the review of studies in the literature evaluating biological activity, safety, and tolerability (70, 71). A previous randomized, crossover trial of NR at 1,000 mg daily for 6 weeks demonstrated improvements in increasing NAD^+^-related metabolites coinciding with improvements in systolic BP (70). At all treatment periods, each participant took the same amount of identical-looking tablets. Participants, study physician, assessors, and study staff were blinded to the treatment and sequence allocation of participants.

Adherence to the study treatment was assessed by phone call from the study coordinator midway through each treatment arm to confirm adherence and inventory method to assess remaining pills at the end of the study. Participants were asked to bring in unused study pills at each visit to return to The University of Washington Investigational Drug Services (IDS), which managed the study drugs. Adherence was defined as consumption of at least 75% of the prescribed regimen.

There were 4 study visits in total for this trial: 1 baseline visit followed by 3 more at the end of each treatment period. The baseline visit was the same as visits 2, 3, and 4, with an addition of medical history and anthropometrics testing. Participants underwent baseline evaluation including a detailed assessment of demographics, smoking history, medical history, medication inventory, and vital signs. Independent functional status was obtained using an ADL score. These were collected using the Lawton-Bowdy Instrumental Activities of Daily Living (IADL) scale (72) coding responses as 0 and 1 summed to a total score of 8, with 8 being the most independent and 0 being the least independent. To avoid introducing any potential bias into the study, the participants were asked to avoid making any changes to their habitual physical activity during the trial. All concomitant medications taken by the participants within 4 weeks prior to study enrollment were recorded. In addition to the prescribed medication, over-the-counter medications (i.e., vitamins, herbal remedies) were also recorded. The participants were also asked to fast at least 6 hours before each visit and abstain from caffeine intake, smoking, and exercising 48 hours before bicycle ergometry testing. Alcohol and recreational or street drug use was also recorded for interpretation and documentation of observed participant health status. These were verified by the study coordinators the day of testing. During each visit, blood and cycle ergometry data were collected for each participant.

A brief screening process to evaluate eligibility criteria and to obtain informed consent took place approximately 1 month before the baseline visit. The screening visit included verification of inclusion and exclusion criteria, measuring vital signs, anthropometrics, 6-minute walking distance, screening labs (renal panel and complete blood count), and bicycle ergometry practice to familiarize participants with the equipment. Qualified participants were randomized to a supplementation sequence (Supplemental Figure 1).

### Study population

Study participants were selected from a larger observational cohort study of muscle energetics in CKD: the Muscle Mitochondrial Energetics and Dysfunction (MEND) study (6). Additional participants were recruited from nephrology clinics at the University of Washington System, including Harborview Medical Center and University of Washington Medical Center. Inclusion criteria were ages 30–79, eGFR of less than 60mL/min/1.73 m^2^ (using the creatinine-based 2012 Chronic Kidney Disease Epidemiology Collaboration equation), and a 6-minute walking distance of less than 550 meters. Exclusion criteria included insulin-dependent diabetes, treatment with dialysis, kidney transplantation, and weight of over 300 lb. A total of 25 participants were recruited for the study (Table 1). The last participant exited the study in April 2021.

### Physical endurance measurements

The primary outcomes of the study were changes in physical exercise endurance measured by aerobic capacity (VO_2_ peak), total work, and work efficiency. We also measured RER via cycle ergometry (Lode Corival Cycle Ergometer, MGC Diagnostics Ultima CardiO_2_ pulmonary exercise system). These measurements were obtained in a dedicated exercise research center at the Fred Hutchinson Cancer Center using cycle ergometry measuring oxygen uptake starting at 0 W at 60 rotations per minute (rpm) and increasing it by 30 W every 3 minutes until exhaustion. If participants were to complete 120 W for 3 minutes, they would continue pedaling at 120 W until exhaustion. A mean rate of perceived exertion (RPE) of greater than 17 (Borg scale) and a mean RER of 1.109 ±.099 were achieved across all visits, suggesting maximal effort was given by the participants (73, 74). The VO_2_ was measured as 30-second averages. The VO_2_ peak was defined as the highest VO_2_ obtained during the last 30 seconds of the exercise testing. Total work performed (kJ) was calculated as follows: (W × seconds)/1,000. Work efficiency during exercise testing was calculated as follows: total work/VO_2_ peak. RER at rest, 30 W, 60 W, and max workload were measured as the last 30-second averaged RER of each specific stage (rest, 30 W, 60 W, and VO_2_ max).

### Metabolic profiling

We evaluated changes in plasma metabolites and circulating lipid species through semitargeted metabolomics and lipidomics profiling approaches using gas chromatography coupled to time-of-flight mass spectrometry (GC TOF-MS) and liquid chromatography coupled to quadrupole time-of-flight mass spectrometer-charged surface hybrid (LC QTOF CHS), respectively, as described before (75, 76). A priori–selected metabolites that use NAD^+^/NADP^+^ as cofactors were targeted for plasma metabolites based on their anticipated response to the study treatments. These metabolites include pyruvate, malate, lactate, isocitrate, glutamate, glucose-6-phosphate, and α-ketoglutarate. Both platforms were performed at West Coast Metabolomics Center. A total of 98 plasma metabolites and 394 lipid species were detected across all 4 visits.

### Statistics

#### Physical endurance.

We used ANOVA to estimate sequence effects, treatment effects, and period effects on physical performance outcomes. The coefficient of the interaction term between the treatment (NR, CoQ10, and placebo) and time point (before versus after) was used to estimate the treatment effect for each intervention. The impact of treatment on BW, kidney function biomarkers, and cystatin C was estimated using the method. To estimate treatment effect in the stratified analysis of physical endurance outcomes, the interaction term of activity level (active versus sedentary) and treatment or performance (poor versus good performers) and treatment were used. Treatment effects in the gender-stratified comparison were estimated using the interaction term of sex (male versus female) and treatment. We tested for possible carryover effects of the study interventions using the interaction of study period and treatment. One-way ANOVA and 2-way ANOVA were performed using GraphPad Prism 9.0. A *P* value of less than 0.05 was considered significant for all analyses unless stated otherwise.

#### Subgroup analysis.

Participants were grouped into the “active” category if they were physically active for at least 300 minutes (moderate intensity) per month, according to their self-reported online survey. This threshold was picked based on the recommendation of the Physical Activity Guidelines for Americans (2nd edition) for our participants’ age demographics with chronic conditions (77). The second grouping strategy was based on participants’ performance during their baseline cycle ergometry testing. The participants who completed 3 minutes at all stages of the exercise protocol from 0 W to 120 W were included, which equals a minimum of 15 minutes total test duration, and were labeled as “good performers.” The participants who were unable to complete the last stage of 120 W and therefore had a test duration less than 15 minutes were labeled “poor performers.”

#### Metabolomics and lipidomics.

Plasma metabolites were available for 25 participants for all 4 visits except for 1 participant who missed a post-CoQ10 visit. All metabolites were checked for normality. To eliminate systematic variation during sample preparation, raw metabolites were normalized using Systematic Error Removal Using Random Forest (SERRF) (78). Differences in plasma metabolites were assessed using a linear mixed modeling approach with random intercepts in which SERRF-normalized metabolites were regressed on treatment type (placebo versus CoQ10 or NR), time point (before versus after), and the interaction of the 2, additionally adjusted for fasting status. Fold changes were calculated using the mean ratio of postsupplementation (CoQ10 or NR) over placebo. To quantify the magnitude of impact on the plasma metabolic and lipid profile, we determined the effect size using standardized mean difference (79). An effect size of 0.5 is considered a medium effect and an effect size above 0.8 is considered a large effect. Linear mixed effects modeling was also used to perform carryover analysis to confirm negligible lingering effects from each supplementation period. Data are presented as mean ± SD unless otherwise stated. Statistical analysis was performed using R 3.6.1 (80).

#### Power calculations.

Sample size was calculated based on a pilot, randomized, controlled trial study of patients with CKD demonstrating the effect of a 12-month combined resistance and aerobic exercise program resulting in an increase of 5.7 mL/kg/min VO_2_ peak with a standard deviation of the difference in VO_2_ peak on bicycle ergometry of 2.1 mL/kg/min (81). The probability is 92% that the study will detect a treatment difference at a 2-sided 0.05 significance level, if the true difference between treatments is 2.1 (1 SD) mL/kg/min. This assumes that the standard deviation of the difference in the response variables is 2.1 mL/kg/min. The same power calculation was employed for each treatment.

### Study approval

The procedures in the study and model informed consent forms were reviewed by the University of Washington Human Subjects Division (HSD). All participants provided written informed consent. The study was approved by the ethical review board of University of Washington (STUDY00004998).

## Author contributions

The conceptualization was contributed by AA, BR, GB, DJM, and BRK. The methodology was contributed by AA, SF, DKP, DJM, BR, and BRK. The formal analysis was conducted by AA and SF. The investigation was performed by BR, BRK, and DKP. Resources were contributed by BRK, BR, JG, and DKP. Data curation was performed by AA, SF, and DKP. The original draft was written by AA and BR. The review was written and edited by AA, BR, GB, APV, JG, JEN, BJB, MPV, BL, DKP, BRK, IHDB, JH, DJM, and BRK. Visualization was contributed by AA and SF. Supervision was carried out by BR, BRK, and DKP. Project administration was contributed by BRK, DKP, IHDB, JH, and BR. Funding acquisition was contributed by BR and BRK.

## Supplementary Material

Supplemental data

ICMJE disclosure forms

## Figures and Tables

**Table 1:**
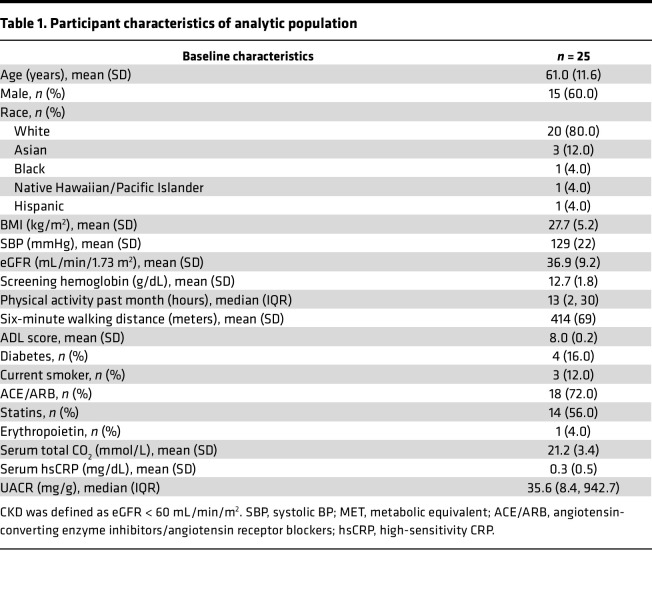
Participant characteristics of analytic population

**Table 2:**
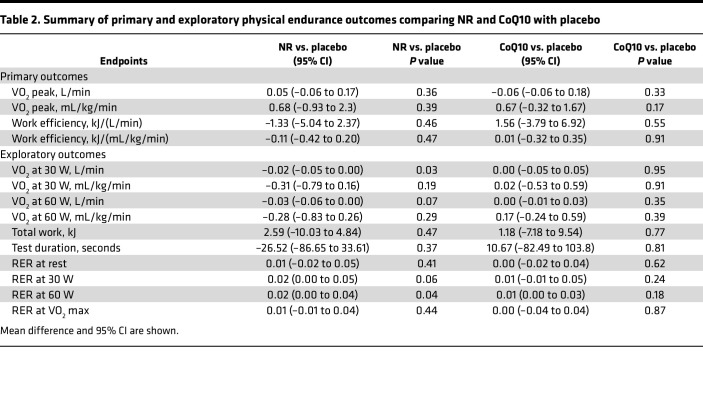
Summary of primary and exploratory physical endurance outcomes comparing NR and CoQ10 with placebo

**Table 3:**
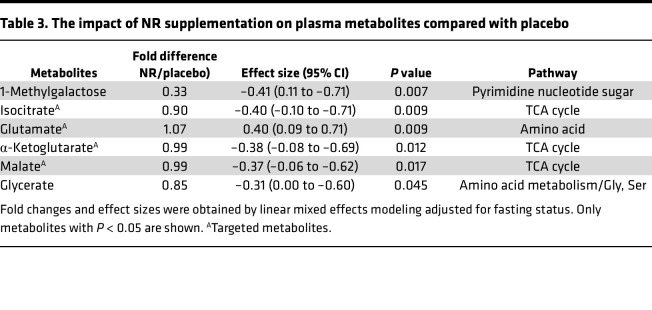
The impact of NR supplementation on plasma metabolites compared with placebo

## Version 1 05/09/2023

In-Press Preview

## Version 2 06/08/2023

Electronic publication

## Version 3 06/27/2023

The last paragraph of the Results section contained a typo. It is fixed now
