# Supplementary material for: Randomized crossover clinical trial of coenzyme Q10 and nicotinamide riboside in chronic kidney disease
Source: JCI Insight. 2023 Jun 8;8(11):e167274. doi: 10.1172/jci.insight.167274 (PMC10393227; doi:10.1172/jci.insight.167274)
Supplement: Supplemental data [file jciinsight-8-167274-s299.pdf]

Supplemental Figure 1. Study design and sample size. Given the efficient crossover design of the study, all 25 participants received each supplement by the end of the study.

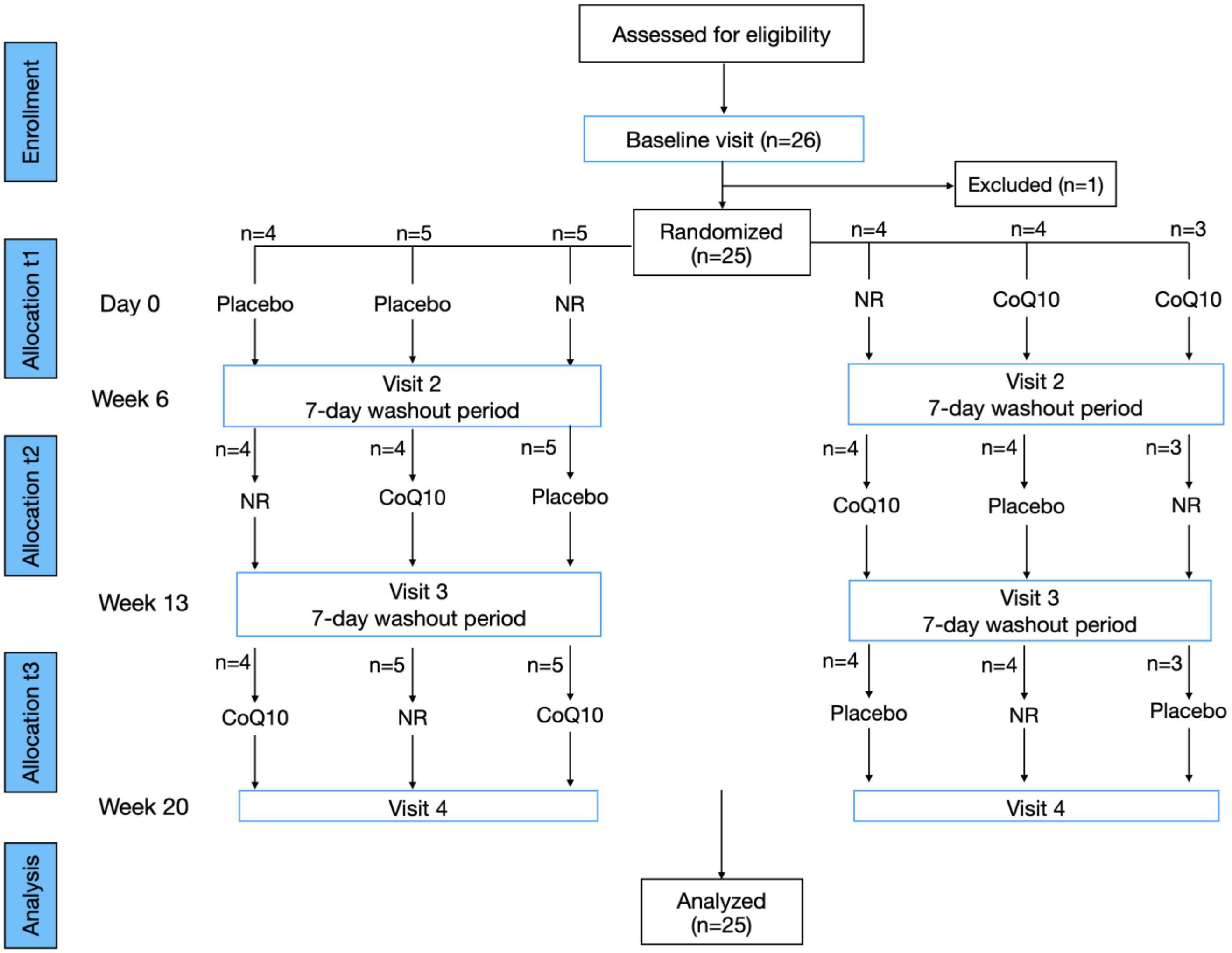

Supplemental Table 1. Estimates of difference in body weight, blood pressure and physical endurance outcomes comparing CoQ10 and NR to placebo. Mean difference and 95% CI are shown.

**Endpoints**	**NR vs. placebo (95% CI)**	**NR vs. placebo P-value**	**CoQ10 vs. placebo (95% CI)**	**CoQ10 vs. placebo P-value**
Body weight, kg	-0.37 (-1.52 to 0.78)	0.51	0.74 (-3.11 to 4.64)	0.69
Systolic blood pressure at 30W, mmHg	5.48 (-4.01 to 14.98)	0.24	3.31 (-5.92 to 12.54)	0.46
Diastolic blood pressure at 30W, mmHg	2.81 (-2.31 to 7.93)	0.26	-1.51 (-5.69 to 2.66)	0.45
Systolic blood pressure at 60W, mmHg	4.32 (-4.29 to 12.95)	0.30	2.62 (-8.32 to 13.58)	0.62
Diastolic blood pressure at 60W, mmHg	3.76 (-1.77 to 9.29)	0.16	-0.33 (-6.22 to 5.55)	0.90
VO_2 peak, L/min	0.05 (-0.06 to 0.17)	0.36	-0.06 (-0.06 to 0.18)	0.33
VO_2 peak, mL/kg/min	0.68 (-0.93 to 2.3)	0.39	0.67 (-0.32 to 1.67)	0.17
VO_2 at 30W, L/min	-0.02 (-0.05 to 0.00)	0.03	0.00 (-0.05 to 0.05)	0.95
VO_2 at 30W, mL/kg/min	-0.31 (-0.79 to 0.16)	0.19	0.02 (-0.53 to 0.59)	0.91
VO_2 at 60W, L/min	-0.03 (-0.06 to 0.00)	0.07	0.00 (-0.01 to 0.03)	0.35
VO_2 at 60W, mL/kg/min	-0.28 (-0.83 to 0.26)	0.29	0.17 (-0.24 to 0.59)	0.39
Total work, kJ	2.59(-10.03 to 4.84)	0.47	1.18(-7.18 to 9.54)	0.77
Work efficiency, kJ/(L/min)	-1.33 (-5.04 to 2.37)	0.46	1.56 (-3.79 to 6.92)	0.55
Work efficiency, kJ/(mL/kg/min)	-0.11(-0.42 to 0.20)	0.47	0.01(-0.32 to 0.35)	0.91
Test Duration, seconds	-26.5 (-86.7 to 33.6)	0.37	10.7 (-82.5 to 103.8)	0.81
RER at rest	0.01(-0.02 to 0.05)	0.41	0.00 (-0.02 to 0.04)	0.62
RER at 30W	0.02 (0.00 to 0.05)	0.06	0.01 (-0.01 to 0.05)	0.24
RER at 60W	0.02(0.00 to 0.04)	0.04	0.01(0.00 to 0.03)	0.18
RER at VO_2 max	0.01 (-0.01 to 0.04)	0.44	0.00 (-0.04 to 0.04)	0.87

Supplemental Figure 2. Stratified analysis of changes in cardiorespiratory fitness and RER at 60W comparing higher vs lower performers and males vs females during cycle ergometry (n=25). (A and C) VO_2 peak and RER at 60W comparison between sedentary (n=9) vs active (n=16) participants. (B and D) VO_2 peak and RER at 60W comparison between good (n=15) vs poor (n=10) performers. (E and F) VO_2 peak and RER at 60W comparison between males (n=15) and females (n=10). Bar graphs represent median (IQR), and the whiskers represent minimum and maximum values. ****P<0.0001,***P <0.001, **P < 0.01, *P < 0.05.

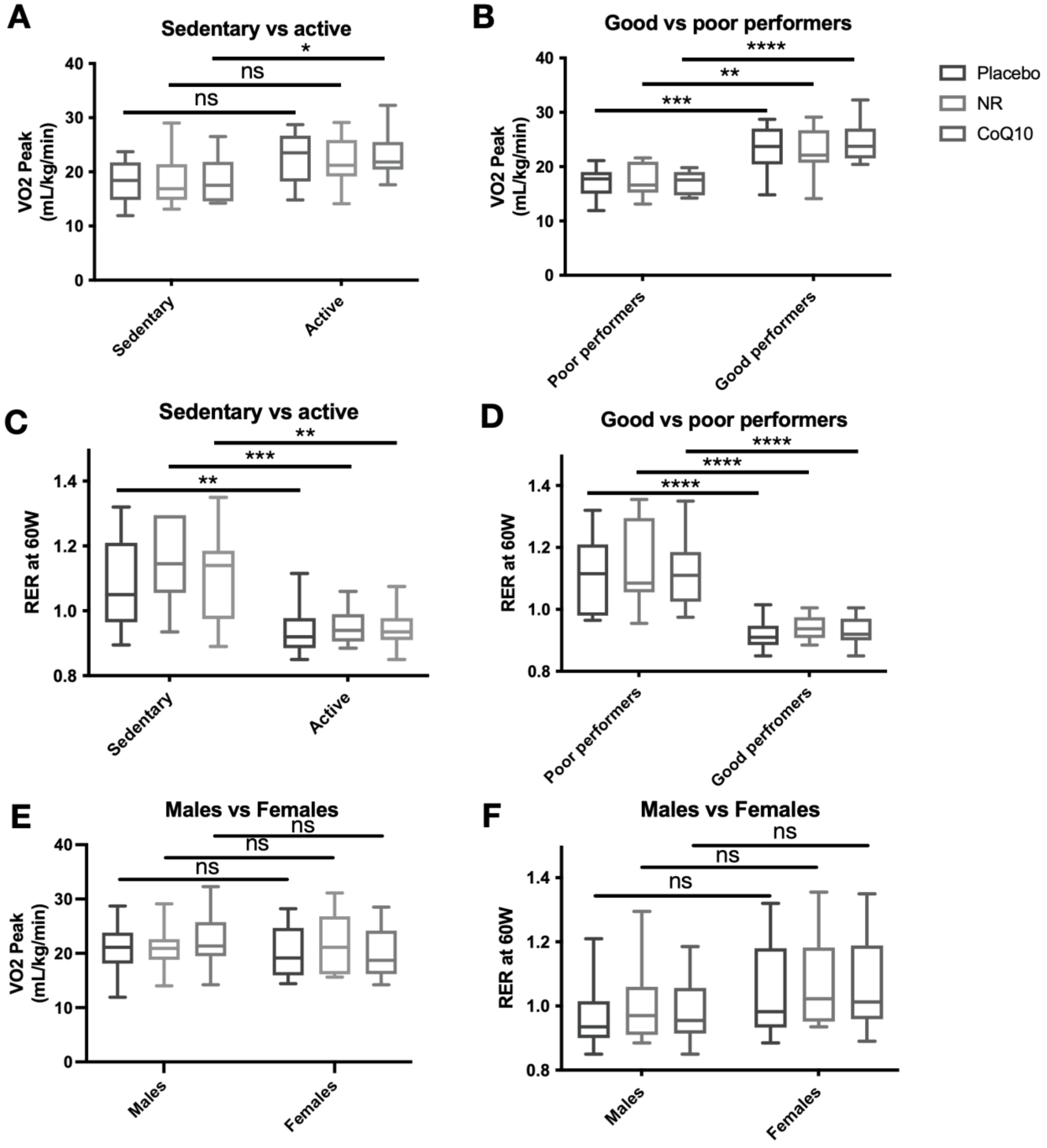

Supplemental Figure 3. Kidney function and inflammatory biomarkers are not impactive by NR or CoQ10 treatment. (A) log-transformed urine albumin to creatinine ratio, (B) serum creatinine, (C) serum cystatin C, and (D) serum C-reactive protein. Bar graph represents mean, and the error bars represent SD for (A and D). Box plots represent median (IQR), and the whiskers represent minimum and maximum values for (B and C).

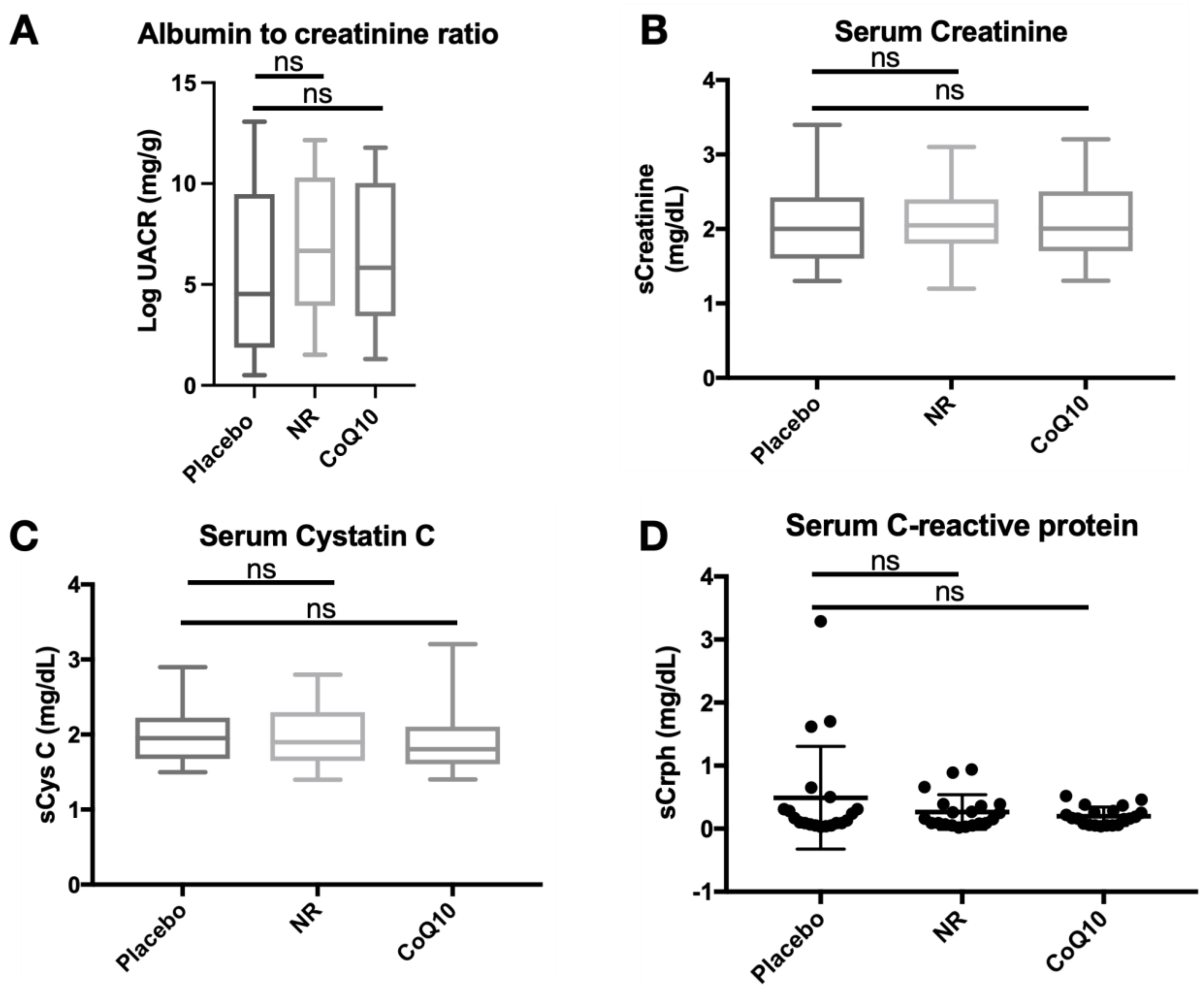

Supplemental Table 2. Differences in plasma lipid profile in response to CoQ10 treatment compared to placebo.

Lipids	Fold difference (CoQ10/placebo)	Effect size (95% CI)	*P*
CE 16:0	1.24	0.53 (0.21 to 0.85)	0.001
TG 59:3	0.60	-0.39 (-0.70 to 0.08)	0.012
TG 54:4	0.78	-0.3 (-0.70 to 0.07)	0.013
PC 30:0	1.26	0.38 (0.07 to 0.68)	0.015
LPE 18:1	0.78	-0.38 (-0.68 0.07)	0.015
FA 18:2 (linoleic acid)	1.40	0.36 (0.05 to 0.66)	0.020
TG 56:4	0.76	-0.36 (-0.66 to -0.05)	0.022
LPC 14:0	1.17	0.35 (0.05 to 0.66)	0.022
PC 31:1	1.20	0.36 (0.04 to 0.66)	0.022
PC 16:0/9:0 CHO	0.87	-0.35 (-0.66 to -0.04)	0.024
FA 20:3 (homo-gamma-linolenic acid)	1.24	0.35 (0.04 to 0.65)	0.024
FA 20:2 (eicosadienoic acid)	1.29	0.35 (0.04 to 0.65)	0.024
CE 18:1	1.14	0.35 (0.04 to 0.66)	0.025
FA 14:1 (physeteric acid)	1.31	0.34 (0.03 to 0.64)	0.029

PC P-36:5 or PC O-36:6	1.26	0.34 (0.02 to 0.64)	0.032
DAG 38:6	0.83	-0.33 (-0.64 to -0.02)	0.033
TG 56:3	0.51	-0.32 (-0.63 to -0.02)	0.036
TG 58:4	0.34	-0.32 (-0.62 to -0.01)	0.038
FA 12:0 (lauric acid)	1.30	0.30 (0.01 to 0.60)	0.045
TG 58:8	0.70	-0.31(-0.62 to -0.01)	0.045
TG 56:6	0.81	-0.31 (-0.61 to 0.00)	0.046
FA 18:1 (oleic acid)	1.35	0.30 (0.00 to 0.60)	0.047
FA 17:0 (margaric acid)	1.19	0.30 (0.00 to 0.60)	0.047
FA 17:1	1.30	0.30 (0.00 to 0.60)	0.047

Fold changes and effect sizes were obtained by linear mixed effects modeling adjusted for fasting status. Only lipids with $P<0.05$ are shown. CE; cholesterol ester, FA; fatty acid, DAG, diacylglycerol; PC, phosphatidylcholine; LPC, lyso-phosphatidylcholine; LPE, lyso-phosphatidylethanolamine; TG, triacylglycerol.

Supplemental Table 3. The impact of NR supplementation on plasma lipid profile compared to placebo.

Lipids	Fold difference (NR/placebo)	Effect size (95% CI)	*P*
CE 18:1	1.22	0.53 (0.21 to 0.85)	0.001
CE 16:0	1.24	0.53 (0.20 to 0.84)	0.001
TG 49:1	0.62	-0.42 (-0.73 to -0.11)	0.007
LPE 20:4	0.86	-0.42 (-0.73 to -0.10)	0.008
TG 51:1	0.66	-0.41(-0.72 to -0.10)	0.009
TG 49:2	0.72	-0.40 (-0.71 to -0.09)	0.011
LPE 18:1	0.77	-0.39 (-0.69 to -0.08)	0.013
TG 54:1	0.56	-0.38 (-0.69 to -0.07)	0.014
Cer d42:0	0.85	-0.38 (-0.69 to -0.07)	0.016
PE 38:5	0.83	-0.38(-0.68 to -0.06)	0.016
TG 52:1	0.59	-0.37 (-0.68 to -0.06)	0.017
Cer d40:0	0.86	-0.37 (-0.67 to -0.05)	0.019
TG 54:2	0.60	-0.36 (-0.67 to -0.05)	0.021
TG 51:2	0.73	-0.36 (-0.66 to -0.04)	0.022
TG 50:0	0.48	-0.35 (-0.65 to -0.04)	0.024

Cer d34:0	0.82	-0.35 (-0.65 to -0.04)	0.026
Cer d44:1	0.91	-0.35 (-0.65 to -0.03)	0.027
DG 38:6	0.82	-0.34 (-0.65 to -0.03)	0.027
LPE 22:6	0.88	-0.34 (-0.65 to -0.03)	0.027
Cer d34:1	0.92	-0.33 (-0.64 to -0.02)	0.032
PC P-44:4 or PC O-44:5	1.07	0.32 (0.01 to 0.63)	0.037
GlcCer d38:1	0.92	-0.32 (-0.63 to -0.01)	0.037
PE 38:4 Isomer B	0.87	-0.32 (-0.63 to -0.01)	0.038
PE 34:1	0.79	-0.32 (-0.62 to -0.01)	0.039
TG 48:3	0.70	-0.31(-0.62 to -0.01)	0.041
LPC 18:0	0.89	-0.31(-0.62 to -0.01)	0.042
LPC 22:4	0.71	-0.31(-0.61 to -0.01)	0.042
PC P-36:5 or PC O-36:6	1.24	0.31 (0.00 to 0.62)	0.046
LPC 18:1	0.90	-0.30 (-0.61 to -0.00)	0.047
TG 49:3	0.77	-0.31 (-0.61 to -0.01)	0.048

Fold changes and effect sizes were obtained by linear mixed effects modeling adjusted for fasting status. Only lipids with $P<0.05$ are shown. CER; ceramide, DAG, diacylglycerol; PE; phosphatidylethanolamine; LPC, Lyso-phosphatidylcholine; LPE, lyso-phosphatidylethanolamine; TG, triacylglycerol.

Supplemental Table 4. Treatment-emergent adverse events ordered by total frequency (n=25). Treatment emergent adverse events were counted from the start of treatment until the end of the washout period.

Adverse events	CoQ10	NR	Placebo
Upper respiratory illness	1 (4%)	1 (4%)	1 (4%)
Nausea	0 (0%)	1 (4%)	1 (4%)
Atrial fibrillation	1 (4%)	0 (0%)	0 (0%)
Cramping	0 (0%)	1 (4%)	0 (0%)
Hyperglycemia	0 (0%)	1 (4%)	0 (0%)
Hypotension	0 (0%)	0 (0%)	1 (4%)
Palpitations	0 (0%)	1 (4%)	0 (0%)
Right bundle branch block	1 (4%)	0 (0%)	0 (0%)
Stiff neck	0 (0%)	1 (4%)	0 (0%)
Swollen arm	0 (0%)	0 (0%)	1 (4%)
